# *IFNL4*-ΔG is associated with prostate cancer among men at increased risk of sexually transmitted infections

**DOI:** 10.1038/s42003-018-0193-5

**Published:** 2018-11-14

**Authors:** Tsion Zewdu Minas, Wei Tang, Cheryl J. Smith, Olusegun O. Onabajo, Adeola Obajemu, Tiffany H. Dorsey, Symone V. Jordan, Obadi M. Obadi, Bríd M. Ryan, Ludmila Prokunina-Olsson, Christopher A. Loffredo, Stefan Ambs

**Affiliations:** 1 Laboratory of Human Carcinogenesis, Center for Cancer Research, National Cancer Institute (NCI), National Institutes of Health (NIH), Bethesda, MD USA; 2 Laboratory of Translational Genomics, Division of Cancer Epidemiology and Genetics, NCI, NIH, Bethesda, MD USA; 3 Cancer Prevention and Control Program, Lombardi Comprehensive Cancer Center, Georgetown University Medical Center, Washington, DC USA

## Abstract

Sexually transmitted infections can reach the prostate gland where their harmful effects are mediated by innate immunity, including interferons. Humans are polymorphic for the germline dinucleotide variant, rs368234815-TT/ΔG, in the *IFNL4* gene encoding interferon λ4. Since the *IFNL4-*ΔG allele has been linked to impaired viral clearance, we hypothesized that potential exposure to sexually transmitted pathogens, as assessed by the number of lifetime sexual partners, may increase prostate cancer risk in an *IFNL4-*ΔG-dependent manner. Accordingly, we find that men with 10 or more sexual partners and at least one copy of *IFNL4-*ΔG have a significantly increased risk of prostate cancer while those with the same number of partners but lacking *IFNL4-*ΔG do not. Moreover, a test for effect modification shows a positive interaction between the number of lifetime partners and *IFNL4-*ΔG in the development of aggressive prostate cancer. Based on these findings, we conclude that a gene–environment interaction between *IFNL4-*ΔG and sexual activity may increase the risk of prostate cancer.

## Abstract (web summary)

Tsion Zewdu Minas et al. find that men carrying an *IFNL4* variant that is more common in African Americans and is associated with impaired viral clearance have a higher risk of prostate cancer. Specifically, this risk is increased in *IFNL4* variant carriers with more lifetime sexual partners, suggesting a link to impaired clearance of sexually transmitted infections

## Introduction

Cancer-causing sexually transmitted infections (STI), such as human papillomavirus (HPV), human immunodeficiency virus (HIV), and herpes simplex virus (HSV), are common worldwide. HPV infections cause cervical and head and neck cancers, while HIV infections cause Kaposi’s sarcoma^1^. The prostate gland is an organ that is easily exposed to infections of the urogenital tract. The innate immune response of the urogenital epithelium provides the first line of defense against pathogens and may protect against urogenital cancers. Accordingly, studies have reported an association between prostate cancer risk and mutations or genetic variants in innate immune response genes^2,3^. However, the evidence for an association between STI and prostate cancer remains inconclusive. Multiple epidemiological studies have evaluated the association between lifetime number of sexual partners, as a proxy for STI, and prostate cancer risk and reported both positive^4–11^ and null associations^12–15^. Therefore, we hypothesized that discordance in these observations could be due to residual confounding by unknown host factors modifying the relationship between STI and prostate cancer.

One of the host factors of importance for innate immune response to STI could be a germline dinucleotide polymorphism, rs368234815 (TT or ΔG alleles), in the *IFNL4* gene that encodes IFN-λ4, a type-III interferon^16,17^. The ΔG allele creates an open reading frame for IFN-λ4, thus this interferon can only be produced in carriers of the ΔG allele. Paradoxically, although IFN-λ4 is an antiviral cytokine and a potent inducer of the JAK-STAT pathway, individuals carrying the *IFNL4*-ΔG allele have impaired ability to clear certain viral infections such as hepatitis C virus (HCV)^16,18,19^, cytomegalovirus^20,21^, coronavirus^22,23^, and possibly HIV^24^. This effect has been attributed to increased induction of negative regulators of interferon signaling by IFN-λ4 that could affect the function of other interferons^16,17^. We recently showed that *IFNL4*-ΔG is closely associated with the occurrence of an interferon signature, termed Interferon-related DNA Damage Resistance Signature (IRDS), in prostate tumors of African-American patients with decreased overall survival in this patient group^25^. Thus, *IFNL4*-ΔG may have a significant function in prostate cancer biology.

In the present study, we identified a gene–environment interaction between *IFNL4*-ΔG and an increased likelihood of exposure to STI resulting in the development of aggressive prostate cancer, suggesting a yet unidentified relationship between an infectious agent and prostate cancer in men with *IFNL4*-ΔG.

## Results

### Clinical and demographic characteristics of participants

Characteristics of the participants in the NCI-Maryland Prostate Cancer Case-Control Study have been previously described^26^. The study enrolled 976 cases (489 African-American [AA] and 487 European-American [EA]) and 1034 population controls (486 AA and 548 EA) (Table 1) who were asked about their sexual history (Supplementary Table 1). Cases and controls had similar age and BMI distributions. More cases, compared to controls, had a first degree family history of prostate cancer (11% vs. 7%, *P* *=* 0.003, Chi-square test), whereas fewer cases than controls had a college or graduate-level degree (32% vs. 50%, *P* *<* 0.001, Chi-square test). Additionally, more cases were current smokers than controls (25% vs. 15%, *P* *<* 0.001, Chi-square test). Among the 976 cases, 138 men (14%) had advanced disease (stage 3 or 4 prostate cancer). High Gleason score (>7) was reported in 167 patients (17%). Both AA and EA men had similar Gleason score distribution, but AA men tended to have higher blood PSA levels at diagnosis. A greater proportion of EA (16%) men, compared with AA men (13%), presented with an advanced disease in this cohort. Based on the combination of low/high-grade and localized/advanced disease (see Methods), 732 and 244 cases were assigned into groups of nonaggressive and aggressive prostate cancer, respectively.

**Table 1:** Characteristics of study population

	Cases^a^			Population controls		
Demographics	All (*n* = 976)	AA (*n* = 489)	EA (*n* = 487)	All (*n* = 1034)	AA (*n* = 486)	EA (*n* = 548)
Age^b^						
Median (IQR) in years	64 (11)	63 (10)	65 (11)	65 (12)	64 (10)	66 (13)
BMI						
Mean (SD) in kg m^−2^	28.2 (4.9)	28.3 (5.5)	28.2 (4.4)	29.0 (5.3)	29.8 (5.5)	28.3 (5.0)
Education, *N* (%)						
High school or less	357 (37)	231 (47)	126 (26)	250 (24)	145 (30)	105 (19)
Some college	301 (31)	172 (35)	129 (26)	268 (26)	144 (30)	124 (23)
College	176 (18)	58 (12)	118 (24)	262 (25)	105 (21)	157 (29)
Graduate	141 (14)	27 (6)	114 (23)	253 (25)	91 (19)	162 (29)
Did not provide	1 (<1)	1 (<1)	–	1 (<1)	1 (<1)	–
*Baseline health factors*						
Family history of prostate cancer^c^, *N* (%)						
No	873 (89)	440 (90)	433 (89)	963 (93)	455 (94)	508 (93)
Yes	103 (11)	49 (10)	54 (11)	71 (7)	31 (6)	40 (7)
Smoking status^d^, *N* (%)						
Current	245 (25)	169 (34)	76 (16)	151 (15)	98 (20)	53 (10)
Former	399 (41)	179 (37)	220 (45)	466 (45)	201 (42)	265 (49)
Never	324 (33)	136 (28)	188 (39)	408 (40)	184 (38)	224 (41)
Did not provide	8 (1)	5(1)	3 (<1)	9 (<1)	3 (<1)	6 (<1)
Stage^e^, *N* (%)						
T1	187 (19)	77 (16)	110 (23)			
T2	651 (67)	351 (72)	300 (62)			
T3	79 (8)	27 (6)	52 (11)			
T4	59 (6)	34 (7)	25 (5)			
Gleason score, *N (%)*						
≤7	809 (83)	404 (83)	405 (83)			
>7	167 (17)	85 (17)	82 (17)			
Disease aggressiveness, *N* (%)						
Nonaggressive disease^f^	732 (75)	372 (76)	360 (74)			
Aggressive disease^g^	244 (25)	117 (24)	127 (26)			
PSA						
Median (IQR) in ng per ml	6.3 (6.0)	7 (7.7)	6 (4.7)			

*AA* African-American, *EA* European-American, *IQR* interquartile range, *SD* standard deviation, *PSA* prostate specific antigen

^a^Cases recruited within 2 years after disease diagnosis with an average interval between diagnosis and enrollment of 6.7 months

^b^Age at study interview

^c^First-degree relative with prostate cancer

^d^Smoking status describes cigarette smoking

^e^Pathologically confirmed using American Joint Committee on Cancer (AJCC) 7th edition

^f^Cases with pathologically confirmed T1 or T2 and Gleason score ≤ 7

^g^Cases with pathologically confirmed T3 or T4 or Gleason score > 7

### Number of sexual partners as proxy to assess exposure to STI

To establish the validity of our surrogate variable (see Methods), we tested if the number of self-reported sexual partners can be used to infer exposure to gonorrhea, an established STI. As shown in Supplementary Table 2, men with 10 or more sexual partners in their teens to 30s were about 3- to 5-fold more likely to have had a gonorrhea infection than men that had only 0–1 sexual partners, while men with 2–9 partners had a 2- to 3-fold increased odds of this infection. These relationships were statistically significant in an analysis of all men combined or when we stratified into controls and cases. The lifetime number of sexual partners also correlated with the likelihood of contracting gonorrhea in an exposure-dependent manner (Supplementary Table 2), and the likelihood of contracting gonorrhea was increased with a higher lifetime number of sexual partners (*P*_trend_ *<* 0.001), further validating the utility of this proxy variable to infer likelihood of exposure to STI.

### Number of sexual partners and prostate cancer risk

As the youngest age at diagnosis in our cohort was 42 years, and 24 of our participants had a prostate cancer diagnosis in their 40s and therefore could not report any sexual partners in their 50s and beyond, we decided to only look at exposures during their teens, 20, and 30s. Table 2 presents the associations of sexual activity during early adulthood (up to age 39) and throughout lifetime with prostate cancer. We did not observe a significant association between the number of sexual partners when the men were teens and their risk of developing prostate cancer later in life, according to the multivariable model, although a trend towards an increased risk was observed in the univariate analysis. On the other hand, when considering the number of sexual partners during an individual’s 20s and 30s, individuals who had 10 or more sexual partners had a 41% and 59% increased odd of prostate cancer, respectively, compared with men who had 0–1 partners. Similarly, when considering the total number of sexual partners through the life course, men who had 20 or more sexual partners had a 47% increased odd of prostate cancer. These associations were significant in the multivariable model and were also dose-dependent. For example, men who had 10 or more sexual partners had more risk than those who had 2–9 sexual partners during their 20s (*P*_trend_ = 0.029) and 30s (*P*_trend_ = 0.001). Our findings indicate that increased exposure to STI during early adulthood elevates individuals’ risk of developing prostate cancer later in life, suggesting that STI may play a role in the etiology of prostate cancer. These findings were observed in both EA and AA men. However, we noted a more robust risk association among the AA men (Table 2).

**Table 2:** Association between number of sexual partners and prostate cancer risk in 976 cases and 1034 controls stratified by race/ethnicity

	Total					African-American					European-American				
	Control *N* (%)	Case *N* (%)	Univariable OR (95% CI)	Multivariable^a^ OR (95% CI)	*P* value^c^	Control *N* (%)	Case *N* (%)	Univariable OR (95% CI)	Multivariable^b^ OR (95% CI)	*P* value^c^	Control *N* (%)	Case *N* (%)	Univariable OR (95% CI)	Multivariable^b^ OR (95% CI)	*P* value^c^
When you were in your teens with how many different partners did you have intercourse?															
0–1	444 (46)	347 (38)	Ref.	Ref.		137 (30)	113 (24)	Ref.	Ref.		307 (60)	234 (54)	Ref.	Ref.	
2–9	458 (47)	456 (51)	1.27 (1.05, 1.54)	1.05 (0.84, 1.32)	0.666	272 (60)	281 (60)	1.25 (0.93, 1.69)	0.96 (0.68, 1.36)	0.823	186 (36)	175 (40)	1.23 (0.94, 1.61)	1.15 (0.84, 1.57)	0.372
10 or more	66 (7)	99 (11)	1.92 (1.36, 2.70)	1.32 (0.89, 1.95)	0.162	45 (10)	71 (15)	1.91 (1.22, 3.00)	1.24 (0.75, 2.07)	0.399	21 (4)	28 (6)	1.75 (0.97, 3.16)	1.31 (0.67, 2.54)	0.429
			*P*_trend_ 0.000	*P*_trend_ 0.229				*P*_trend_ 0.006	*P*_trend_ 0.530				*P*_trend_ 0.028	*P*_trend_ 0.284	
When you were in your 20s with how many different partners did you have intercourse?															
0–1	308 (31)	243 (26)	Ref.	Ref.		80 (17)	56 (12)	Ref.	Ref.		228 (42)	187 (39)	Ref.	Ref.	
2–9	526 (53)	485 (51)	1.17 (0.95, 1.44)	1.14 (0.89, 1.46)	0.291	281 (61)	277 (58)	1.41 (0.96, 2.06)	1.45 (0.94, 2.23)	0.092	245 (45)	208 (44)	1.04 (0.79, 1.35)	1.04 (0.77, 1.40)	0.816
10 or more	167 (17)	222 (23)	1.68 (1.30, 2.19)	**1.41 (1.04, 1.92)**	**0.027**	100 (22)	141 (30)	2.01 (1.31, 3.09)	**1.82 (1.12, 2.97)**	**0.015**	67 (12)	81 (17)	1.47 (1.01, 2.15)	1.27 (0.82, 1.97)	0.282
			*P*_trend_ 0.000	*P*_trend_ **0.029**				*P*_trend_ 0.001	***P***_**trend**_ **0.017**				*P*_trend_ 0.085	*P*_trend_ 0.345	
When you were in your 30s with how many different partners did you have intercourse?															
0–1	544 (54)	412 (43)	Ref.	Ref.		158 (34)	102 (21)	Ref.	Ref.		386 (71)	310 (65)	Ref.	Ref.	
2–9	354 (35)	402 (42)	1.50 (1.24, 1.82)	**1.39 (1.11, 1.76)**	**0.005**	226 (49)	274 (58)	1.88 (1.38, 2.55)	**1.73 (1.22, 2.46)**	**0.002**	128 (24)	128 (27)	1.25 (0.93, 1.66)	1.16 (0.84, 1.60)	0.368
10 or more	103 (10)	137 (14)	1.76 (1.32, 2.34)	**1.59 (1.14, 2.22)**	**0.007**	77 (17)	99 (21)	1.99 (1.35, 2.94)	**1.79 (1.15, 2.78)**	**0.010**	26 (5)	38 (8)	1.82 (1.08, 3.06)	1.66 (0.93, 2.96)	0.084
			*P*_trend_ 0.000	***P***_**trend**_ **0.001**				*P*_trend_ 0.000	***P***_**trend**_ **0.005**				*P*_trend_ 0.012	*P*_trend_ 0.078	
Throughout your life, what is the total number of partners with whom you have had sexual intercourse?															
<5	347 (34)	260 (27)	Ref.	Ref.		79 (17)	56 (12)	Ref.	Ref.		268 (50)	204 (43)	Ref.	Ref.	
5–19	416 (41)	388 (40)	1.24 (1.01, 1.54)	1.14 (0.88, 1.46)	0.320	220 (46)	211 (44)	1.35 (0.92, 2.00)	1.30 (0.83, 2.03)	0.253	196 (36)	(37)	1.19 (0.90, 1.56)	1.08 (0.78, 1.48)	0.646
20 or more	254 (25)	315 (33)	1.66 (1.31, 2.08)	**1.47 (1.11, 1.95)**	**0.007**	177 (37)	216 (45)	1.72 (1.16, 2.56)	1.57 (0.99, 2.48)	0.053	77 (14)	99 (21)	1.69 (1.19, 2.39)	**1.58 (1.06, 2.35)**	**0.025**
			*P*_trend_ 0.000	***P***_**trend**_ **0.006**				*P*_trend_ 0.005	***P***_**trend**_ **0.046**				*P*_trend_ 0.004	***P***_**trend**_ **0.040**	

*OR* odds ratio, *95% CI* 95% confidence interval

^a^Unconditional logistic regression adjusted for body mass index at study enrollment (BMI, kg m^−2^), age at study entry, education (high school or less, some college, college, professional school), family history of prostate cancer (first degree relatives,

yes/no), smoking history (never, former, current), condom use (usually use, yes/no), aspirin use (regular user, yes/no), IFNL4 rs368234815 genotype (ΔG/ΔG or ΔG/TT vs. TT/TT), and race

^b^Unconditional logistic regression adjusted for body mass index at study enrollment (BMI, kg m^−2^), age at study entry, education (high school or less, some college, college, professional school), family history of prostate cancer (first degree relatives,

yes/no), smoking history (never, former, current), condom use (usually use, yes/no), aspirin use (regular user, yes/no), and IFNL4 rs368234815 genotype (ΔG/ΔG or ΔG/TT vs. TT/TT)

Bolded data indicate significant associations in the multivariable logistic regression analysis

^c^Bonferroni-corrected significance thresholds for multiple comparisons in the total (*n* = 4) and stratified (*n* = 8) analyses are 0.0125 and 0.00625, respectively

### *IFNL4*-ΔG modifies sexual activity-associated cancer risk

**Fig. 1:**
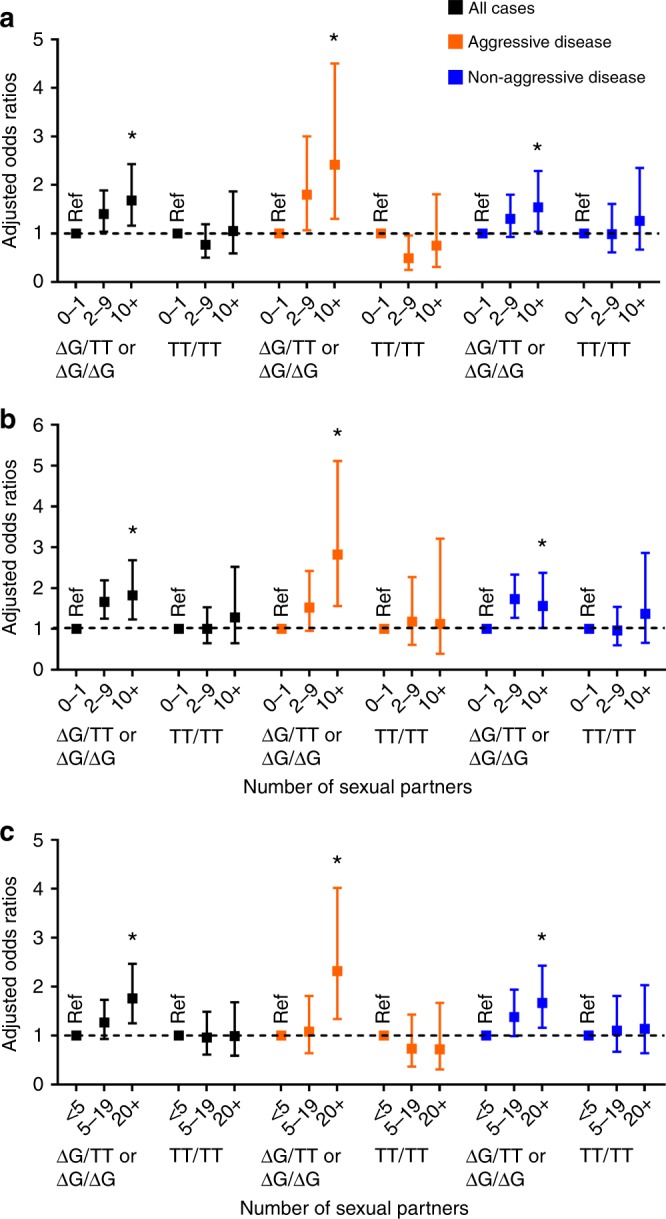
Summary odds ratios with 95% confidence intervals for the relationship between the number of sexual partners and prostate cancer with stratification by *IFNL4* genotypes. The number of sexual partners is significantly associated with prostate cancer only among carriers of the *IFNL4*-ΔG allele (ΔG/TT or ΔG/ΔG). Shown are the odds ratios for all prostate cancer patients combined, for patients with aggressive disease or nonaggressive disease. **a** Number of sexual partners in the 20s. **b** Number of sexual partners in the 30s. **c** Number of lifetime sexual partners. *P_trend_ < 0.05; Ref. reference group. Adjusted odds ratios as described in Tables. The error bars represent 95% confidence intervals

*IFNL4* rs368234815 genotypes were determined for 828 cases and 953 controls with available germline DNA. As expected^16^, the ΔG allele was more common among AA (61.7%) than EA (33.7%) men (Supplementary Table 3). To investigate whether *IFNL4*-ΔG modifies the association between likelihood of exposure to STI and prostate cancer risk, we stratified the participants into those with one or two ΔG alleles vs. none. As shown in Fig. 1 and Table 3, the adjusted logistic regression analysis revealed that the increased risk of prostate cancer associated with sexual activity was restricted to carriers of at least one ΔG allele. For instance, among individuals that had 10 or more sexual partners in their 20s, the odds of diagnosis with prostate cancer for men with at least one ΔG allele vs. none was 1.68-fold (95% CI: 1.16, 2.43) vs. 1.05-fold (95% CI: 0.59, 1.87), respectively. The effect of the ΔG allele remained statistically significant after adjusting for race/ethnicity or applying a Bonferroni-adjusted significance threshold of *P* < 0.00625.

**Table 3:** Association between number of sexual partners and prostate cancer risk in 828 cases and 953 controls stratified by IFNL4 rs368234815 genotype

	Total					IFNL4 ΔG/TT or ΔG/ΔG					IFNL4 TT/TT				
	Control *N* (%)	Case *N* (%)	Univariable OR (95% CI)	Multivariable^a^ OR (95% CI)	*P* value^c^	Control *N* (%)	Case *N* (%)	Univariable OR (95% CI)	Multivariable^b^ OR (95% CI)	*P* value^c^	Control *N* (%)	Case *N* (%)	Univariable OR (95% CI)	Multivariable^b^ OR (95% CI)	*P* value^c^
When you were in your teens with how many different partners did you have intercourse?															
0–1	444 (46)	347 (38)	Ref.	Ref.		250 (41)	186 (35)	Ref.	Ref.		152 (54)	96 (43)	Ref.	Ref.	
2–9	458 (47)	456 (51)	1.27 (1.05, 1.54)	1.05 (0.84, 1.32)	0.666	312 (51)	286 (53)	1.23 (0.96, 1.58)	1.01 (0.76, 1.33)	0.968	113 (40)	110 (49)	1.54 (1.07, 2.22)	1.19 (0.78, 1.81)	0.415
10 or more	66 (7)	99 (11)	1.92 (1.36, 2.70)	1.32 (0.89, 1.95)	0.162	47 (8)	65 (12)	1.86 (1.22, 2.83)	1.37 (0.87, 2.16)	0.175	17 (6)	19 (8)	1.77 (0.88, 3.57)	1.23 (0.56, 2.70)	0.599
			*P*_trend_ 0.000	*P*_trend_ 0.229				*P*_trend_ 0.004	*P*_trend_ 0.311				*P*_trend_ 0.014	*P*_trend_ 0.414	
When you were in your 20s with how many different partners did you have intercourse?															
0–1	308 (31)	243 (26)	Ref.	Ref.		181 (29)	116 (21)	Ref.	Ref.		96 (33)	78 (32)	Ref.	Ref.	
2–9	526 (53)	485 (51)	1.17 (0.95, 1.44)	1.14 (0.89, 1.46)	0.291	334 (53)	310 (55)	1.45 (1.09, 1.92)	**1.40 (1.04, 1.89)**	**0.029**	153 (52)	121 (49)	0.97 (0.66, 1.43)	0.77 (0.50, 1.19)	0.234
10 or more	167 (17)	222 (23)	1.68 (1.30, 2.19)	**1.41 (1.04, 1.92)**	**0.027**	115 (18)	136 (24)	1.85 (1.31 (2.59)	**1.68 (1.16, 2.43)**	**0.006**	43 (15)	46 (19)	1.32 (0.79, 2.20)	1.05 (0.59, 1.87)	0.858
			*P*_trend_ 0.000	*P*_trend_ **0.029**				*P*_trend_ 0.000	*P*_trend_ **0.006**				*P*_trend_ 0.389	*P*_trend_ 0.923	
When you were in your 30s with how many different partners did you have intercourse?															
0–1	544 (54)	412 (43)	Ref.	Ref.		326 (52)	211 (38)	Ref.	Ref.		172 (59)	131 (53)	Ref.	Ref.	
2–9	354 (35)	402 (42)	1.50 (1.24, 1.82)	**1.39 (1.11, 1.76)**	**0.005**	229 (36)	260 (46)	1.75 (1.37, 2.25)	**1.66 (1.25, 2.19)**	**0.000**	99 (34)	89 (36)	1.18 (0.82, 1.70)	1.00 (0.65, 1.53)	0.993
10 or more	103 (10)	137 (14)	1.76 (1.32, 2.34)	**1.59 (1.14, 2.22)**	**0.007**	74 (12)	91 (16)	1.90 (1.34, 2.70)	**1.82 (1.23, 2.68)**	**0.003**	22 (8)	26 (11)	1.55 (0.84, 2.86)	1.28 (0.65, 2.52)	0.468
			*P*_trend_ 0.000	*P*_trend_ **0.001**				*P*_trend_ 0.000	*P*_trend_ **0.000**				*P*_trend_ 0.133	*P*_trend_ 0.592	
Throughout your life, what is the total number of partners with whom you have had sexual intercourse?															
<5	347 (34)	260 (27)	Ref.	Ref.		199 (31)	131 (23)	Ref.	Ref.		113 (39)	84 (34)	Ref.	Ref.	
5–19	416 (41)	388 (40)	1.24 (1.01, 1.54)	1.14 (0.88, 1.46)	0.320	276 (43)	231 (41)	1.27 (0.96, 1.68)	1.27 (0.93, 1.73)	0.127	110 (38)	92 (37)	1.13 (0.76, 1.67)	0.96 (0.61, 1.49)	0.843
20 or more	254 (25)	315 (33)	1.66 (1.31, 2.08)	**1.47 (1.11, 1.95)**	**0.007**	171 (26)	208 (36)	1.85 (1.37, 2.49)	**1.76 (1.25, 2.47)**	**0.001**	70 (24)	70 (28)	1.35 (0.87, 2.08)	0.99 (0.59, 1.68)	0.980
			*P*_trend_ **0.000**	*P*_trend_ **0.006**				*P*_trend_ 0.000	*P*_trend_ **0.001**				*P*_trend_ 0.185	*P*_trend_ 0.996	

^a^Unconditional logistic regression adjusted for body mass index at study enrollment (BMI, kg m^−2^), age at study entry, education (high school or less, some college, college, professional school), family history of prostate cancer (first degree relatives, yes/no), smoking history (never, former, current), condom use (usually use, yes/no), aspirin use (regular user, yes/no), race, and IFNL4 rs368234815 genotype (ΔG/ΔG or ΔG/TT vs. TT/TT)

^b^Unconditional logistic regression adjusted for body mass index at study enrollment (BMI, kg m^−2^), age at study entry, education (high school or less, some college, college, professional school), family history of prostate cancer (first degree relatives, yes/no), smoking history (never, former, current), condom use (usually use, yes/no), aspirin use (regular user, yes/no), and race

Bolded data indicate significant associations in the multivariable logistic regression analysis

^c^Bonferroni-corrected significance thresholds for multiple comparisons in the total (*n* = 4) and stratified (*n* = 8) analyses are 0.0125 and 0.00625, respectively

We reasoned that among men who had multiple sexual partners during early adulthood, carriers of two copies of ΔG allele (ΔG/ΔG genotype) would be expected to have a higher prostate cancer risk than those with one copy (ΔG/TT genotype). To increase the statistical power to test this hypothesis, we combined individuals with moderate likelihood of exposure (2–9 sexual partners) with the highest likelihood of exposure (10+sexual partners) and compared their risk to the reference group (0–1 sexual partners). As shown in Supplementary Table 4, among those with an increased likelihood of exposure to STI in their 20s, 30s, or during their lifetime, the odds of diagnosis with prostate cancer were higher in carriers of ΔG/ΔG than of ΔG/TT or TT/TT genotypes. Again, there was no significant relationship between the number of sexual partners and prostate cancer risk among TT/TT carriers. However, ΔG/ΔG carriers who had two or more sexual partners in their 20s and 30s, or had 20 or more sexual partners during their lifetime, had increased risk. For instance, for men with two or more sexual partners vs. none during their 30s, the odds ratios increased from 1.05 (95% CI: 0.79, 1.57) to 1.62 (95% CI: 1.16, 2.27) to 1.86 (95% CI: 1.17, 2.95) if their *IFNL4* genotypes were TT/TT, ΔG/TT, or ΔG/ΔG, respectively. Similarly, among men who had 20 or more sexual partners vs. <5 partners in their lifetime, the odds ratios increased from 1.00 (95% CI: 0.59, 1.68) to 1.64 (95% CI: 1.06, 2.53) to 1.97 (95% CI: 1.10, 3.51) if their *IFNL4* genotypes were TT/TT, ΔG/TT, or ΔG/ΔG, respectively.

To further examine this observation, we tested for statistical significance of an interaction term between *IFNL4*-ΔG and likelihood of exposure to STI during early adulthood, using the number of sexual partners as the surrogate measurement for STI likelihood. This analysis was performed for all prostate cancer cases and then stratified into nonaggressive and aggressive disease. Notably, the strongest evidence for a gene–environment interaction revealed an association of this interaction with the odds of developing the aggressive disease (Table 4), where both *IFNL4*-ΔG and multiple lifetime sexual partners synergistically increased the odds for aggressive prostate cancer (*P* interaction = 0.004). This association remained significant when a Bonferroni-adjusted significance threshold of *P* < 0.00625 was applied. We made similar observations when we examined the association between number of sexual partners and disease risk among *IFNL4*-ΔG carriers in strata of aggressive and nonaggressive prostate cancer. As shown in Fig. 1 and Table 5, *IFNL4*-ΔG carriers who had 10 or more sexual partners in their 20s and 30s (or 20 or more lifetime partners), tended to have higher odds of being diagnosed with an aggressive than a nonaggressive disease.

**Table 4:** Test for synergy between *IFNL4* rs368234815 and number of sexual partners in their association with prostate cancer

	All prostate cancer cases		Nonaggressive prostate cancer		Aggressive prostate cancer	
	OR (95% CI)	*P* interaction^a^	OR (95% CI)	*P* interaction^a^	OR (95% CI)	*P* interaction^a^
Number of sexual partners when in teens		0.583		0.357		0.353
0–1 * ΔG/ΔG or ΔG/TT	Ref.		Ref.		Ref.	
2–9 * ΔG/ΔG or ΔG/TT	0.87 (0.54, 1.38)		0.76 (0.46, 1.26)		1.22 (0.58, 2.59)	
10+ * ΔG/ΔG or ΔG/TT	1.12 (0.47, 2.65)		0.83 (0.33, 2.07)		2.80 (0.63, 12.50)	
Number of sexual partners when in 20s		0.077		0.729		**0.003**
0–1 * ΔG/ΔG or ΔG/TT	Ref.		Ref.		Ref.	
2–9 * ΔG/ΔG or ΔG/TT	**1.75 (1.06, 2.89)**		1.25 (0.72, 2.16)		**3.60 (1.63, 7.96)**	
10+* ΔG/ΔG or ΔG/TT	1.58 (0.83, 3.01)		1.19 (0.59, 2.41)		**3.32 (1.21, 9.09)**	
Number of sexual partners when in 30s		0.128		0.068		0.188
0–1 * ΔG/ΔG or ΔG/TT	Ref.		Ref.		Ref.	
2–9 * ΔG/ΔG or ΔG/TT	1.53 (0.97, 2.43)		1.56 (0.94, 2.58)		1.38 (0.66, 2.87)	
10+ * ΔG/ΔG or ΔG/TT	1.29 (0.61, 2.70)		0.97 (0.44, 2.17)		2.66 (0.84, 8.40)	
Lifetime number of sexual partners		0.172		0.598		**0.004**
<5 * ΔG/ΔG or ΔG/TT	Ref.		Ref.		Ref.	
5–19 * ΔG/ΔG or ΔG/TT	1.30 (0.78, 2.16)		1.23 (0.70, 2.17)		1.44 (0.64, 3.23)	
20+ * ΔG/ΔG or ΔG/TT	1.64 (0.94, 2.85)		1.32 (0.72, 2.42)		**3.04 (1.27, 7.24)**	

*P* values for departure from additive interaction based on likelihood ratio tests between the models with and without interaction terms.

Analyses were adjusted for body mass index at study enrollment (BMI, kg m^−2^), age at study entry, education (high school or less, some college, college, professional school), family history of prostate cancer (first degree relatives, yes/no), smoking history (never, former, current) condom use (usually use, yes/no), aspirin use (regular user, yes/no), and race

Bolded data indicate statistical evidence for synergy

^a^Bonferroni-corrected significance thresholds for multiple comparisons in the total (*n* = 4) and stratified (*n* = 8) analyses are 0.0125 and 0.00625, respectively

**Table 5:** Association between number of sexual partners and risk for aggressive prostate cancer vs. nonaggressive prostate cancer stratified by IFNL4 rs368234815 genotype

	IFNL4 ΔG/TT or ΔG/ΔG							IFNL4 TT/TT						
	Control* N* (%)	Aggressive case * N* (%)	Nonaggressive case * N* (%)	Aggressive case Multivariable^a^ OR (95% CI)	*P* value^b^	Nonaggressive case Multivariable^a^ OR (95% CI)	*P* value^b^	Control *N* (%)	Aggressive case* N* (%)	Nonaggressive case* N* (%)	Aggressive case Multivariable^a^ OR (95% CI)	*P* value^b^	Nonaggressive case Multivariable^a^ OR (95% CI)	*P* value^b^
When you were in your teens with how many different partners did you have intercourse?														
0–1	250 (41)	41 (31)	145 (36)	Ref.		Ref.		152 (54)	28 (47)	68 (41)	Ref.		Ref.	
2–9	312 (51)	75 (56)	211 (52)	1.38 (0.86, 2.21)	0.187	0.92 (0.68, 1.24)	0.563	113 (40)	27 (46)	83 (50)	1.19 (0.62, 2.28)	0.607	1.20 (0.75, 1.90)	0.447
10 or more	47 (8)	18 (13)	47 (12)	1.82 (0.90, 3.66)	0.095	1.24 (0.76, 2.03)	0.388	17 (6)	4 (7)	15 (9)	0.72 (0.18, 2.92)	0.648	1.43 (0.62, 3.29)	0.400
				*P*_trend_ 0.074		*P*_trend_ 0.716					*P*_trend_ 0.977		*P*_trend_ 0.324	
When you were in your 20s with how many different partners did you have intercourse?														
0–1	181 (29)	26 (19)	90 (21)	Ref.		Ref.		96 (33)	29 (43)	49 (28)	Ref.		Ref.	
2–9	334 (53)	80 (57)	230 (55)	**1.80 (1.07, 3.00)**	**0.026**	1.30 (0.93, 1.80)	0.123	153 (52)	27 (40)	94 (53)	**0.49 (0.25, 0.96)**	**0.036**	0.99 (0.61, 1.61)	0.978
10 or more	115 (18)	34 (24)	102 (24)	**2.42 (1.30, 4.50)**	**0.005**	**1.54 (1.04, 2.29)**	**0.032**	43 (15)	12 (18)	34 (19)	0.75 (0.31, 1.81)	0.524	1.26 (0.67, 2.35)	0.474
				***P***_**trend**_ **0.005**		***P***_**trend**_ **0.032**					*P*_trend_ 0.253		*P*_trend_ 0.527	
When you were in your 30s with how many different partners did you have intercourse?														
0–1	326 (52)	53 (38)	158 (37)	Ref.		Ref.		172 (59)	38 (55)	93 (53)	Ref.		Ref.	
2–9	229 (36)	57 (41)	203 (48)	1.52 (0.95, 2.42)	0.078	**1.73 (1.27, 2.33)**	**0.000**	99 (34)	24 (35)	65 (37)	1.18 (0.61, 2.27)	0.630	0.96 (0.60, 1.54)	0.873
10 or more	74 (12)	30 (21)	61 (14)	**2.82 (1.56, 5.11)**	**0.001**	**1.56 (1.02, 2.38)**	**0.040**	22 (8)	7 (10)	19 (11)	1.12 (0.39, 3.21)	0.827	1.37 (0.66, 2.86)	0.395
				***P***_**trend**_ **0.001**		***P***_**trend**_ **0.005**					*P*_trend_ 0.694		*P*_trend_ 0.575	
Throughout your life, what is the total number of partners with whom you have had sexual intercourse?														
<5	199 (31)	39 (27)	92 (22)	Ref.		Ref.		113 (39)	29 (43)	55 (31)	Ref.		Ref.	
5–19	276 (43)	44 (31)	187 (44)	1.08 (0.64, 1.81)	0.783	1.38 (0.99, 1.94)	0.058	110 (38)	23 (34)	69 (39)	0.73 (0.37, 1.43)	0.358	1.10 (0.67, 1.81)	0.704
20 or more	171 (26)	60 (42)	148 (35)	**2.32 (1.34, 4.02)**	**0.003**	**1.67 (1.16, 2.43)**	**0.006**	70 (24)	16 (24)	54 (30)	0.72 (0.31, 1.67)	0.450	1.14 (0.64, 2.03)	0.661
				***P***_**trend**_ **0.001**		***P***_**trend**_ **0.007**					*P*_trend_ 0.403		*P*_trend_ 0.653	

*OR* odds ratio, *95% CI* 95% confidence interval

^a^Unconditional logistic regression adjusted for body mass index at study enrollment (BMI, kg m^−2^), age at study entry, education (high school or less, some college, college, professional school), family history of prostate cancer (first degree relatives, yes/no), smoking history (never, former, current), condom use (usually use, yes/no), aspirin use (regular user, yes/no), and race

Bolded data indicate significant associations in the multivariable logistic regression analysis

^b^Bonferroni-corrected significance threshold for multiple comparisons (*n* = 16) is 0.003125

*IFNL4*-ΔG has very divergent allelic frequencies across populations—<10% in Asians, ~30% in Europeans, and up to 78% in Africans^16^—which can lead to a wide range of population attributable risks associated with this genetic variant. Given that the allele frequency is higher in AA, we stratified our analysis by race/ethnicity. We found that the odds of being diagnosed with aggressive prostate cancer in men who had 10 or more sexual partners in their 20s or 30s (or 20 or more lifetime partners) was higher among AA men than EA men (Table 6), consistent with the increased frequency of the *IFNL4*-ΔG risk allele in AA compared to any other racial group. For AA men, having multiple sexual partners (10 or more) in their 30s, increased their odds of diagnosis with aggressive prostate cancer by 207% vs. only by 43% for nonaggressive prostate cancer (Table 6). Similarly, in men with at least one copy of the ΔG allele, having multiple sexual partners in their 30s was associated with 182% increased odds of a diagnosis with aggressive prostate cancer vs. only 56% for nonaggressive prostate cancer (Table 5). In EA men, however, these relationships were either non-significant, or borderline significant, again suggesting that the higher prevalence of the ΔG allele in AA men could contribute to their increased risk for aggressive prostate cancer.

**Table 6:** Association between number of sexual partners and risk for aggressive prostate cancer vs. nonaggressive prostate cancer stratified by race/ethnicity

	African-American							European-American						
	Control *N* (%)	Aggressive case* N* (%)	Nonaggressive case* N* (%)	Aggressive case Multivariable^a^ OR (95% CI)	*P* value^b^	Nonaggressive case Multivariable^a^ OR (95% CI)	*P* value^b^	Control *N* (%)	Aggressive Case * N* (%)	Nonaggressive case * N* (%)	Aggressive case Multivariable^a^ OR (95% CI)	*P* value^b^	Nonaggressive case Multivariable^a^ OR (95% CI)	*P* value^b^
When you were in your teens with how many different partners did you have intercourse?														
0–1	137 (30)	24 (21)	89 (25)	Ref.		Ref.		307 (60)	58 (52)	176 (54)	Ref.		Ref.	
2–9	272 (60)	69 (62)	212 (60)	1.16 (0.63, 2.12)	0.641	0.89 (0.61, 1.30)	0.548	186 (36)	46 (41)	129 (40)	1.33 (0.81, 2.19)	0.253	1.10 (0.78, 1.55)	0.586
10 or more	45 (10)	19 (17)	52 (15)	1.30 (0.57, 2.97)	0.534	1.18 (0.69, 2.03)	0.541	21 (4)	7 (6)	21 (6)	1.58 (0.57, 4.39)	0.377	1.27 (0.62, 2.62)	0.512
				*P*_trend_ 0.522		*P*_trend_ 0.747					*P*_trend_ 0.198		*P*_trend_ 0.449	
When you were in your 20s with how many different partners did you have intercourse?														
0–1	80 (17)	14 (12)	42 (12)	Ref.		Ref.		228 (42)	55 (44)	132 (38)	Ref.		Ref.	
2–9	281 (61)	61 (54)	216 (60)	1.40 (0.67, 2.90)	0.373	1.44 (0.90, 2.30)	0.126	245 (45)	54 (44)	154 (44)	1.06 (0.66, 1.69)	0.808	1.07 (0.77, 1.50)	0.678
10 or more	100 (22)	38 (34)	103 (29)	**2.42 (1.08, 5.40)**	**0.031**	1.64 (0.97, 2.78)	0.063	67 (12)	15 (12)	66 (19)	1.04 (0.50, 2.16)	0.914	1.41 (0.88, 2.26)	0.148
				***P***_**trend**_ **0.018**		*P*_trend_ 0.078					*P*_trend_ 0.852		*P*_trend_ 0.188	
When you were in your 30s with how many different partners did you have intercourse?														
0–1	158 (34)	24 (21)	78 (22)	Ref.		Ref.		386 (71)	82 (66)	228 (65)	Ref.		Ref.	
2–9	226 (49)	58 (51)	216 (60)	1.59 (0.86, 2.91)	0.136	**1.76 (1.21, 2.55)**	**0.003**	128 (24)	33 (27)	95 (27)	1.28 (0.78, 2.11)	0.334	1.14 (0.80, 1.63)	0.461
10 or more	77 (17)	32 (28)	67 (19)	**3.07 (1.52, 6.21)**	**0.002**	1.43 (0.88, 2.32)	0.151	26 (5)	9 (7)	29 (8)	1.28 (0.51, 3.24)	0.596	**1.89 (1.02, 3.50)**	**0.042**
				***P***_**trend**_ **0.002**		*P*_trend_ 0.072					*P*_trend_ 0.339		*P*_trend_ 0.059	
Throughout your life, what is the total number of partners with whom you have had sexual intercourse?														
<5	79 (17)	17 (15)	39 (11)	Ref.		Ref.		268 (50)	62 (50)	142 (40)	Ref.		Ref.	
5–19	220 (46)	43 (37)	168 (46)	1.20 (0.56, 2.56)	0.633	1.34 (0.83, 2.18)	0.232	196 (36)	37 (30)	140 (39)	0.81 (0.48, 1.36)	0.427	1.23 (0.87, 1.74)	0.238
20 or more	177 (37)	57 (49)	159 (43)	1.91 (0.90, 4.06)	0.093	1.46 (0.89, 2.40)	0.133	77 (14)	25 (20)	74 (21)	1.61 (0.88, 2.96)	0.122	**1.66 (1.07, 2.56)**	**0.023**
				***P***_**trend**_ **0.043**		*P*_trend_ 0.167					*P*_trend_ 0.275		***P***_**trend**_ **0.023**	

*OR* odds ratio, *95% CI* 95% confidence interval

^a^Unconditional logistic regression adjusted for body mass index at study enrollment (BMI, kg m^−2^), age at study entry, education (high school or less, some college, college, professional school), family history of prostate cancer (first degree relatives, yes/no),

smoking history (never, former, current), condom use (usually use, yes/no), aspirin use (regular user, yes/no), and IFNL4 rs368234815 genotype (ΔG/ΔG or ΔG/TT vs. TT/TT)

Bolded data indicate significant associations in the multivariable logistic regression analysis

^b^Bonferroni-corrected significance threshold for multiple comparisons (*n*=16) is 0.003125.

### Endogenous IFN-λ4 is induced by viral infection in PC3 cells

**Fig. 2:**
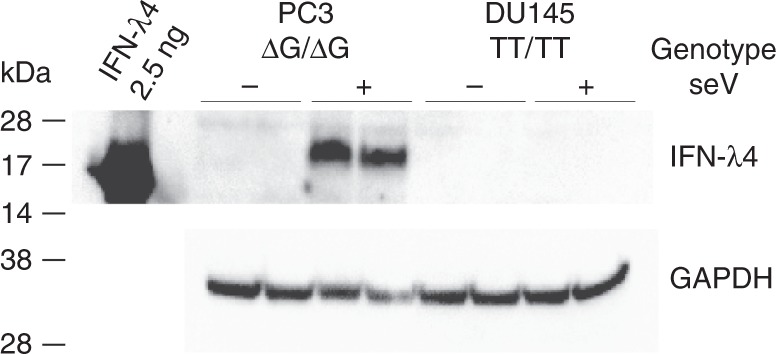
Infection of human prostate cancer cell lines with Sendai virus (SeV) induces expression of IFN-λ4 protein. PC3 and DU145 cells were infected with SeV for 24 h, and induction of IFN-λ4 was examined by western blotting with an anti-IFN-λ4 antibody. Expression of GAPDH was used as a loading control. Shown are results of biological duplicates of cells non-infected (−) and infected (+) with SeV. Recombinant purified IFN-λ4 (2.5 ng) was used as a positive control; glycosylation of endogenous IFN-λ4 produced in mammalian cells increases its molecular weight compared to non-glycosylated recombinant protein produced in *E. coli*. IFN-λ4 expression is detected in PC3 cells (ΔG/ΔG genotype) but not in DU145 cells (TT/TT genotype). Original full-size versions of Western blot images are presented in Supplementary Fig. 2

Because the exact viral pathogens causing STI in the prostate gland are unknown, we infected human prostate cancer cell lines with Sendai virus (SeV), which is a murine RNA virus that infects diverse human cell types and induces a robust antiviral response, including IFN-λ4 expression in primary human hepatocytes^17^. In SeV-infected human prostate cancer cell lines, PC3 and DU145, we observed a robust induction of the transcripts in the *IFNL4* region (Supplementary Figure 1, Supplementary Table 5). Of note, mRNA induction is not allele-specific and can be detected in cells with the TT/TT genotype, although expression is generally lower with the TT/TT genotype due to nonsense-mediated decay of prematurely terminated out-of-frame transcripts^16^. However, IFN-λ4 protein can be produced only in the presence of the ΔG allele. Indeed, our Western blot analysis showed that IFN-λ4 was strongly induced in the SeV-infected PC3 cells that have the ΔG/ΔG genotype but not in DU145 cells that have the TT/TT genotype (Fig. 2, Supplementary Figure 2).

## Discussion

As a key finding, our study revealed a gene–environment interaction in prostate cancer etiology between increased exposure to STI due to sexual activity and a germline genetic variant, *IFNL4*-ΔG, that regulates the innate immune response to infections in epithelial cells. Our observations were consistent across age group and *IFNL4* genotype strata. Furthermore, the odds of developing prostate cancer, particularly of an aggressive type, increased with the number of sexual partners in men carrying the *IFNL4*-ΔG allele. As AA are more likely than EA men to carry the ΔG allele, the effect was more pronounced in the former population. The identification of this novel gene–environment interaction suggests an association between a yet unidentified infectious agent and prostate cancer risk in men with *IFNL4*-ΔG. Efforts are warranted to identify this etiologic agent.

Since the first documented implication of STI as a possible etiologic agent for prostate cancer in the early 1950s^27^, multiple epidemiologic studies have subsequently explored this relationship. Yet, epidemiologic evidence for the association of STI with prostate cancer is still not firmly established. Multiple studies with self-reported exposure to any STI or to the most common, symptomatic STI (i.e. gonorrhea) have reported a positive association^4,6,8,9,11,14,28–31^, resembling the epidemiologic literature for cervical cancer before the discovery of HPV as the etiologic agent. In our study, we observed that self-reported exposure to gonorrhea increased the odds of diagnosis with prostate cancer by about 30% (Supplementary Table 6), which is in concordance with the 2005^32^ and the 2015^33^ meta-analysis data that reported a 35% and 31% increase in risk, respectively. The search for prostate cancer etiologic agents also encompassed sero-epidemiologic evaluations of the association between prostate cancer risk and infection with human herpesvirus 8, herpes simplex type 2, HPV, *Chlamydia trachomatis*, and *Trichomonas vaginalis*, but the reported findings are far from conclusive^34–45^. Although our observations of a gene–environment interaction related to a STI do not implicate a specific STI, they suggest that exposure to some sexually transmitted etiologic agent(s) may lead to prostate cancer, but only in genetically susceptible individuals.

In our NCI-Maryland case-control study, we observed that men who had multiple sexual partners, and hence may have had an increased likelihood of exposures to STI during early adulthood, had higher odds of diagnosis with aggressive prostate cancer later in life. Consistent with the increased prevalence of the ΔG allele among individuals of African ancestry, this association was stronger in AA than EA men. In vitro infection of prostate cancer cell lines with SeV, a model for a wide range of RNA viruses, showed induction of IFN-λ4 in a cell line homozygote for the ΔG allele (PC3) but not in a cell line without this allele (DU145), supporting the hypothesis for a viral origin of IFN-λ4 expression in prostate tissue. Until now, a detectable induction of endogenous IFN-λ4 protein was only shown for hepatic cells^16,17^. Once induced, IFN-λ4 protein may enhance negative regulation of IFN signaling, leading to impaired viral clearance and a distinct tumor biology in carriers of *IFNL4-*ΔG allele, as previously reported^16,17,25^. An impaired IFN signaling may also affect innate immune response to non-viral pathogens.

*IFNL4*-ΔG is more common in individuals of African ancestry (up to 78%), compared with those of European ancestry (~30%)^16^, and we observed a comparable distribution in our case-control study, with 61.7% and 33.7% *IFNL4*-ΔG allele frequencies among AA and EA men, respectively (Supplementary Table 3). This is in line with the health disparity in prostate cancer that manifests as a more aggressive and deadly disease in AA compared to other racial groups. Moreover, the findings of this study are also consistent with our previous work where we observed a distinct interferon signature that was more prevalent in tumors from AA than EA men^25,46^. A role of the interferon-inducible system in the pathogenesis of an aggressive form of prostate cancer is further suggested by germline mutations in *RNASEL*, a gene involved in the interferon antiviral pathway, that were found to predispose men to familial prostate cancer^2,47^.

Carriers of the *IFNL4*-ΔG allele who had multiple sexual partners during early adulthood had about a 3-fold increase in their odds of being diagnosed with an aggressive prostate cancer later in life. On the contrary, men with the *IFNL4*-TT variant did not bear a similar STI-associated prostate cancer risk even if they had as many partners. However, *IFNL4*-ΔG may not predispose to prostate cancer in the context of all STI. For example, it has been shown that a herpesvirus infection and its latency confer protection from bacterial infections^48^. Thus, chronic viral infection in carriers of *IFNL4*-ΔG may lead to a decreased risk for a bacterial infection. Accordingly, we found that the association of gonorrhea with prostate cancer was stronger for *IFNL4*-TT/TT than *IFNL4*-ΔG carriers (Supplementary Table 6), arguing that the overall unfavorable association of *IFNL4*-ΔG with prostate cancer, as shown in our study, is more likely related to an infectious agent of viral origin. Additional studies are needed to identify this microbial agent.

Our study has several limitations that should be considered. In general, case-control study designs are influenced by inherent biases including recall, response, and interviewer biases. Our study should not be affected by interviewer bias since sexual history information was acquired through a self-administered questionnaire filled in privacy. However, we relied on retrospective, self-reported assessment of sexual history, which is vulnerable to recall bias. In our view, it is unlikely that the general population is aware of the infectious hypothesis in the context of prostate cancer, arguing against a recall bias. Additionally, the fact that the self-reported number of sexual partners could be used to infer exposure to gonorrhea in both cases and controls in a similar manner suggests that the case-control status did not influence self-reported sexual history (Supplementary Table 2). Likewise, the fact that we observed STI-associated prostate cancer risks exclusively in men with at least one copy of the ΔG allele, whereas not in men with the TT/TT genotype, albeit both groups having reported multiple sexual partners, makes it rather unlikely that a recall bias contributed to these findings. As we report an association of *IFNL4*-ΔG with disease risk in the context of sexual activity, there could only be confounding/bias in this observation if the men knew their *IFNL4* genotype, which they certainly did not, and then reported sexual activity biased by this knowledge. To further buttress the validity of our observations, we also found a significant relationship between a gonorrhea infection history and prostate cancer in a very consistent manner with recent meta-analyses^32,33^. Yet, it remains a limitation that we could not validate our findings in a second cohort. Our study population is rather unique having AA subjects, an in-depth survey about sexual activity, and germline DNA for genotyping, making it difficult to replicate our findings without the requirement for additional recruitment. Nevertheless, the fact that we observed a dose response relationship that fits a biologically plausible mechanism suggests that the associations we observed warrant further investigations, including prospective cohorts.

In conclusion, an increased likelihood of exposure to STI among men with genetically impaired clearance of viral infections was found to be positively associated with the risk of prostate cancer. Although our findings do not implicate a specific STI, they strongly argue for an infection as an etiologic agent for prostate cancer in a substantial subset of patients. Whether the observed association is due to carcinogenic effects of a putative etiologic infectious agent or rather due to the host’s response to the infectious agent needs to be examined in future work. Our data suggest that the etiology of prostate cancer may vary in population groups due to the ancestry-related genetic variation in resolution or progression of an infection following exposure. The proposed synergistic mechanism of prostate cancer pathophysiology is relevant for all populations but could disproportionally affect men of African ancestry. As such, these findings may shed light on the prostate cancer health disparity.

## Methods

### NCI-Maryland prostate cancer case-control study

This case-control study has been previously described^26^. The study was initiated to test the primary hypothesis that environmental exposures and ancestry-related factors contribute to the excessive prostate cancer burden among AA men. Briefly, prior to interview, all subjects signed informed consent for participation. All study forms and procedures were approved by the NCI (protocol # 05-C-N021) and the University of Maryland (protocol #0298229) Institutional Review Boards. Cases were recruited at the Baltimore Veterans Affairs Medical Center and the University of Maryland Medical Center.

Eligibility criteria included the following: diagnosis with prostate cancer within two years prior to enrollment, residence in Maryland or adjacent counties in Pennsylvania, Delaware, Virginia, or District of Columbia, 40 to 90 years old at the time of enrollment, born in the United States, either African-American (AA) or European-American (EA) by self-report, can be interviewed in English, had a working home phone number, physically and mentally fit to be interviewed, not severely ill, and not residing in an institution such as prison, nursing home, or shelter. A total of 976 cases (489 AA and 487 EA men) were recruited into the study between 2005 and 2015.

Controls were identified through the Maryland Department of Motor Vehicle Administration database and were frequency-matched to cases on age and race. The controls also had the same eligibility criteria as cases with the exception that they could not have a personal history of cancer (other than non-melanoma skin cancer), radiation therapy, or chemotherapy. A total of 1034 population controls were recruited (486 AA and 548 EA men). At the time of enrollment, both cases and controls were administered a survey by a trained interviewer and a blood sample or mouthwash rinse/buccal cells was collected. The survey asked about their demographics, tobacco use, nutrition, medical history, family history of cancer, prostatitis, or benign prostatic hypertrophy, occupational history, socioeconomic status, anthropometry, and sexual history. The participants were given 20 min of privacy to complete the sexual history section of the survey.

### Assessment of sexual history

The sexual history section of the survey assessed the participants’ number of sexual partners using the following questions: (1) Throughout your life, what is the total number of partners with whom you have had sexual intercourse—fewer than 5, 5 to 9, 10 to 19, 20 to 30, 40 or more; (2) when you were (in your teens, in your 20s, in your 30s, in your 40s, in your 50s, in your 60s, or in your 70s) with how many different partners did you have intercourse—0, 1, 2, 3–4, 5–9, 10–19, 20–39, 40 or more. More than 90% of the participants (92%, 97%, 97%, and 99% of the cases and 94%, 97%, 97%, and 98% of the controls) reported the number of sexual partners they had in their teens, 20s, 30s, and throughout their life, respectively; the remainder either declined to answer or provided incomplete information (Supplementary Table 1). For lifetime number of sexual partners, the “5 to 9” and “10 to 19” categories were collapsed to a new a category “5 to 19” whereas the “20 to 30” and “40 or more” categories were collapsed together into a new category “20 or more”. Similarly, for the number of sexual partners that the men had during their different age decades (teens, 20s, 30s, 40s, 50s, 60s, 70s), we collapsed the categories to three groups: a reference group (0–1 sexual partners) with presumed no or low chance of contracting a STI, a medium risk group (2–9 sexual partners) with intermediate likelihood of contracting a STI, and a higher risk group (10 or more sexual partners) with the highest likelihood of contracting a STI. The sexual history section also asked about the history of condom use using the following question: do you usually use condoms (rubbers)—no, yes.

### Assessment of history of sexually transmitted disease

The medical history section of the questionnaire asked about the participants’ history of two sexually transmitted diseases using the following questions: (1) Did a doctor ever tell you that you had gonorrhea—no, yes; (2) Did a doctor ever tell you that you had syphilis—no, yes; if the participants answered “yes” to either of the two questions, they were asked the follow-up questions: “How old were you when you were first diagnosed?” and “How many times altogether have you had the disease?” History of syphilis infection was not analyzed in detail because it was reported by only few participants (31 cases and 26 controls). Infection with gonorrhea was more common (226 cases and 172 controls) and this information was used for analysis.

### Prostate cancer classification

Gleason grading system was used to classify prostate tumors into low-grade (Gleason score ≤ 7) or high-grade (Gleason score > 7). Tumor stage at diagnosis was obtained from medical records, stage 1 or 2 was defined as localized and stage 3 or 4 as advanced disease. Cases with a combination of low-grade tumors and localized disease were defined as nonaggressive prostate cancer, while cases with either high-grade tumors or advanced disease were defined as aggressive prostate cancer.

### Genotyping of IFNL4 rs368234815

Genomic DNA was isolated from buffy coats, when available, and from cell lines using the DNeasy Blood & Tissue Kit (Qiagen). For a subset of men (100 cases and 112 controls), genomic DNA was obtained from a mouthwash sample using the Gentra Puregene Buccal Cell Kit (Qiagen). In total, genomic DNA was available for 828 cases (84.8% of study participants) and 953 controls (92.2%). Genotyping of rs368234815 was done as previously described^16^ with a custom-designed assay purchased from Thermo Fisher. The genotyping was performed using ABI 7900 (Applied Biosystems) according to the standard protocol. Genotype concordance among duplicates was 97%. There was no deviation from the Hardy–Weinberg equilibrium in controls. Genotyping success rate was 98.6%. The genotyping failure rate was similar (1.4%) for DNA extracted from blood and mouthwash samples. The genotype along with the clinical information for the 976 cases and 1034 controls is presented in Supplementary Data 1.

### SeV infection and expression analysis

Human prostate cancer cell lines, PC3 (*IFNL4* rs368234815-ΔG/ΔG genotype) and DU145 (TT/TT genotype), were obtained from the American Type Culture Collection (Manassas, VA); the cells have been regularly authenticated using a short tandem repeat analysis with GenePrint10 and tested for Mycoplasma contamination. Both cell lines are not commonly misidentified per the register of misidentified cell lines curated by ICLAC (International Cell Line Authentication Committee). PC3 cells were cultured in F-12K Medium (ATCC, Manassas, VA) with 10% FBS, and DU145 were cultured in Eagle’s Minimum Essential Medium (ATCC) with 10% FBS. SeV infection was done as described^17^. For IFN-λ4 protein expression analysis, the cells were seeded overnight at a density of 0.3 × 10^6^ cells in six-well plates and infected in triplicates with SeV Cantell strain (Charles River Laboratories) or mock (control) for 24 h, harvested, lysed with 100 µl of RIPA (Sigma) supplemented with protease inhibitor (Promega) and pulse sonicated for 30s, with 10s burst-cooling cycles, at 4 °C. Lysates were centrifuged at 10,000 × *g* for 5 min, supernatant was boiled in reducing sample buffer for 5 min at 100 °C, resolved on 4–12% Bis-Tris Bolt gels, and transferred using an iBlot 2 (Thermo Fisher). The blot was blocked with 2% milk for 1 h and then incubated with rabbit anti-IFN-λ4 (1:500, ab196984; Abcam) and developed with secondary HRP-linked goat anti-rabbit antibody (1:5000, #7074; Cell Signaling Technology) and SuperSignal West Femto Maximum Sensitivity Substrate (Thermo Fisher). For gene expression, cells were infected with SeV, as described above, but for 10 h. Total RNA was extracted using an RNeasy Kit with on-column DNase I digestion (Qiagen). cDNA was generated from 500 ng of total RNA with the RT2 First Strand Kit (Qiagen) with an additional DNA removal step. Total RNA input was 2.5 ng per reaction. Expression was measured in technical quadruplicates—of *IFNL4, ISG15*, and endogenous controls, *GAPDH* and *ACTB*, using TaqMan expression assays. SeV loads were measured with a SYBR Green expression assay for SeV defective-interfering RNA, as previously described^17^. QuantStudio 7 (Thermo Fisher) was used for the expression analysis.

### Statistical analysis

All statistical analyses were performed using Stata/SE 14.0 statistical software package (Stata Corp). All statistical tests were two-sided and *P* *<* 0.05 was considered statistically significant. We used the Bonferroni’s method for multiple comparison correction based on the number of survey questions in the main analyses (*n* = 4) and for additional subgroups in the stratified analyses. Bonferroni-adjusted significance thresholds are reported in the footnotes of Tables 2–6. For the main analysis, we evaluated whether the sexual history variables were associated with prostate cancer and then stratified by race/ethnicity (AA vs. EA) and *IFNL4* rs368234815 (ΔG/ΔG or ΔG/TT vs. TT/TT) for all cases or cases with aggressive vs. nonaggressive disease. Unconditional logistic regression was used to compute the odds ratios (OR) and 95% confidence intervals (CI) to assess the association of sexual history and medical history parameters (number of sexual partners during the different age decades, total number of sexual partners, or history of gonorrhea) with prostate cancer. We adjusted for potential confounding factors: body mass index at study enrollment (BMI, kg m^−2^), age at enrollment, education (high school or less, some college, college, professional school), family history of prostate cancer (first degree relatives, yes/no), smoking history (never, former, current), condom use (usually use, yes/no), aspirin use (regular user, yes/no), *IFNL4* rs368234815 (ΔG/ΔG or ΔG/TT vs. TT/TT), and race/ethnicity as applicable.

To test for a gene–environment interaction between *IFNL4*-ΔG and the number of sexual partners as a proxy for likelihood of exposure to STI, we examined for departure from additivity^49^. We applied the multivariable model with and without the interaction term and examined significance with the likelihood ratio test. The results were adjusted for potential confounding factors listed above. A *P* *<* 0.05 was considered as statistical evidence for effect modification.

## Electronic supplementary material

Supplementary file

Description of additional supplementary items

Supplementary Data 1

## Data Availability

Datasets generated during and/or analyzed during the current study are available from the corresponding author on reasonable request. The polymorphism data for *IFNL4* are provided in Supplementary Data 1.
