# Supplementary material for: IFNL4-ΔG is associated with prostate cancer among men at increased risk of sexually transmitted infections
Source: Commun Biol. 2018 Nov 14;1:191. doi: 10.1038/s42003-018-0193-5 (PMC6235841; doi:10.1038/s42003-018-0193-5)
Supplement: Supplementary file 1 — Supplementary file [file 42003_2018_193_MOESM1_ESM.pdf]

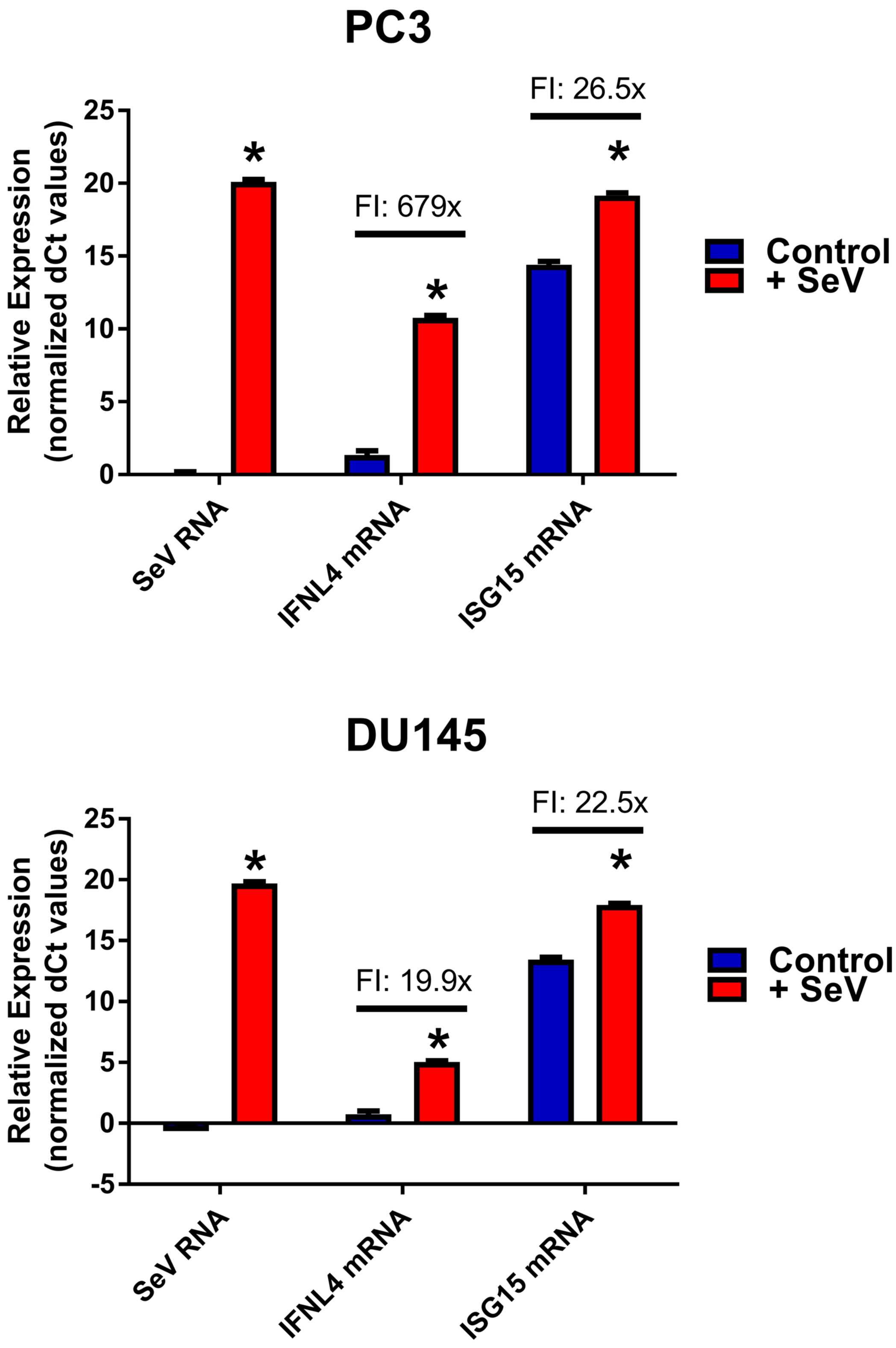

Supplementary Figure 1: Infection of human prostate cancer cell lines with Sendai virus (SeV) induces expression of transcripts in the IFNL4 region. PC3 and DU145 cells were infected with SeV. Induction of IFNL4 and ISG15 was determined by qRT-PCR. ISG15, a known interferon-stimulated gene, was added as a positive control. dCt values for IFNL4 and ISG15 were normalized using expression of endogenous controls, GAPDH and ACTB. Shown are normalized dCt values, log 2 scale, as mean ± SD for gene expression (n=3) in control (not infected) and SeV-infected cells (+ SeV). Viral load (SeV RNA) was determined by qRT-PCR, as described in methods. * $P < 0.001$, two-sided Student's t-test. IFNL4 genotypes in PC3 and DU145 cells are ΔG/ΔG and TT/TT, respectively. For display purposes, normalized dCt values are shown as dCt + 18.2. FI = fold induction. The expression values used for plotting this figure are presented in Supplementary Table 5.

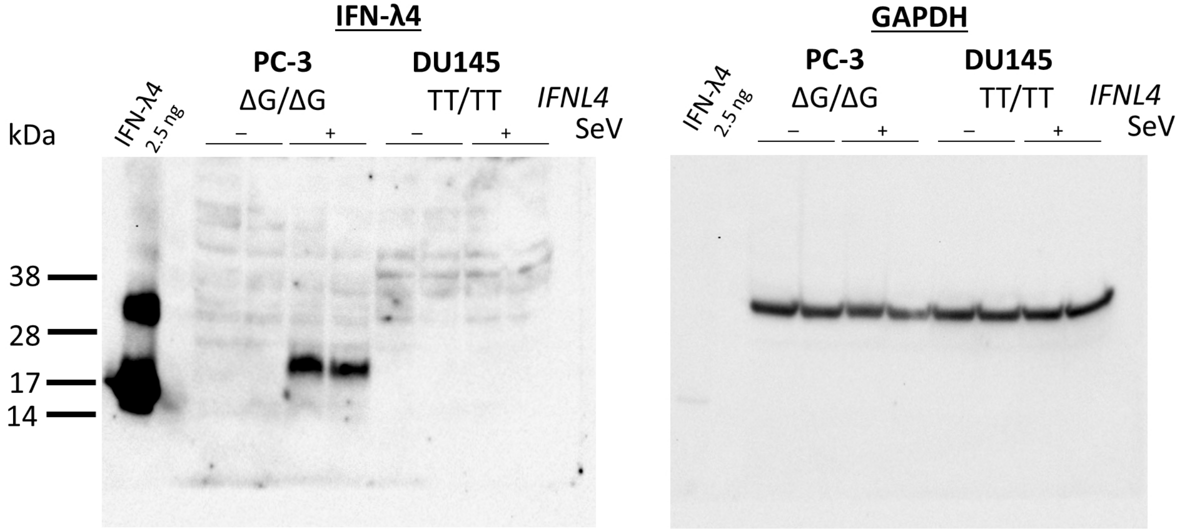

Supplementary Figure 2. Infection of human prostate cancer cell lines with Sendai virus (SeV) induces expression of IFN-λ4 protein. Original full-size versions of Western blot images presented in Figure 2. PC3 and DU145 cells were infected with SeV for 24 hours, and induction of IFN-λ4 was examined by Western blotting with an anti-IFN-λ4 antibody. Expression of GAPDH was used as a loading control. Shown are results of biological duplicates of cells non-infected (-) and infected (+) with SeV. Recombinant purified IFN-λ4 (2.5 ng) was used as a positive control; glycosylation of endogenous IFN-λ4 produced in mammalian cells increases its molecular weight compared to non-glycosylated recombinant protein produced in E. coli. IFN-λ4 expression is detected in PC3 cells (ΔG/ΔG genotype) but not in DU145 cells (TT/TT genotype).

Supplementary Table 1. Sexual history of participants in the NCI Maryland Prostate Cancer Case-Control Study

	**Cases[a]**			**Population Controls**		
	All (n=976)	AA[b] (n=489)	EA[c] (n=487)	All (n=1034)	AA (n=486)	EA (n=548)
**When you were in your teens with how many different partners did you have intercourse?** ***N (%)***						
0-1	347 (36)	113 (23)	234 (48)	444 (43)	137 (28)	307 (56)
2-9	456 (47)	281 (57)	175 (36)	458 (44)	272 (56)	186 (34)
10 or more	99 (10)	71 (15)	28 (6)	66 (6)	45 (9)	21 (4)
Did not provide	74 (8)	24 (5)	50 (10)	66 (6)	32 (7)	34 (6)
**When you were in your 20s with how many different partners did you have intercourse?** ***N (%)***						
0-1	243 (25)	56 (11)	187 (38)	308 (30)	80 (16)	228 (42)
2-9	485 (50)	277 (57)	208 (43)	526 (51)	281 (58)	245 (45)
10 or more	222 (23)	141 (29)	81 (17)	167 (16)	100 (21)	67 (12)
Did not provide	26 (3)	15 (3)	11 (2)	33 (3)	25 (5)	8 (1)
**When you were in your 30s with how many different partners did you have intercourse?** ***N (%)***						
0-1	412 (42)	102 (21)	310 (64)	544 (53)	158 (33)	386 (70)
2-9	402 (41)	274 (56)	128 (26)	354 (34)	226 (47)	128 (23)
10 or more	137 (14)	99 (20)	38 (8)	103 (10)	77 (16)	26 (5)
Did not provide	25 (3)	14 (3)	11 (2)	33 (3)	25 (5)	8 (1)
**Throughout your life, what is the total number of partners with whom you have had sexual intercourse?** ***N (%)***						
<5	260 (27)	56 (11)	204 (42)	347 (34)	79 (16)	268 (49)
5-19	388 (40)	211 (43)	177 (36)	416 (40)	220 (45)	196 (36)
20 or more	315 (32)	216 (44)	99 (20)	254 (25)	177 (36)	77 (14)
Did not provide	13 (1)	6 (1)	7 (1)	17 (2)	10 (2)	7 (1)
**Did a doctor ever tell you that you had gonorrhea?** ***N (%)***						
No	750 (77)	308 (63)	442 (91)	862 (83)	339 (70)	523 (95)
Yes	226 (23)	181 (37)	45 (9)	172 (17)	147 (30)	25 (5)

[a]Cases recruited within 2 years after disease diagnosis with an average interval between diagnosis and enrollment of 6.7 months

[b] AA: African-American

[c] EA: European American

Supplementary Table 2. Association between number of sexual partners and likelihood of contracting gonorrhea

	Total			Controls			Cases		
	Did a doctor ever tell you that you had gonorrhea?			Did a doctor ever tell you that you had gonorrhea?			Did a doctor ever tell you that you had gonorrhea?		
	**No** *N* **(%)**	**Yes** *N* **(%)**	**Multivariable[a] OR (95% CI)**	**No** *N* **(%)**	**Yes** *N* **(%)**	**Multivariable[a] OR (95% CI)**	**No** *N* **(%)**	**Yes** *N* **(%)**	**Multivariable[a] OR (95% CI)**
**When you were in your teens with how many different partners did you have intercourse?**									
0-1	718 (48)	73 (19)	Ref.	409 (51)	35 (22)	Ref.	309 (45)	38 (18)	Ref.
2-9	675 (45)	239 (63)	**2.15 (1.54, 2.99)**	350 (43)	108 (67)	**2.40 (1.49, 3.88)**	325 (47)	131 (60)	**1.84 (1.16, 2.94)**
10 or more	98 (7)	67 (18)	**3.97 (2.51, 6.28)**	47 (6)	19 (12)	**2.85 (1.38, 5.89)**	51 (7)	48 (22)	**4.75 (2.57, 8.78)**
			P $_{trend}$ ***<0.000***			***P*** $_{trend}$ ***<0.000***			***P*** $_{trend}$ ***<0.000***
**When you were in your 20s with how many different partners did you have intercourse?**									
0-1	520 (33)	31 (8)	Ref.	292 (35)	16 (10)	Ref.	228 (31)	15 (7)	Ref.
2-9	801 (51)	210 (55)	**2.45 (1.58, 3.79)**	432 (52)	94 (57)	**2.38 (1.30, 4.37)**	369 (51)	116 (52)	**2.50 (1.32, 4.75)**
10 or more	245 (16)	144 (37)	**5.43 (3.40, 8.68)**	113 (14)	54 (33)	**5.23 (2.70, 10.16)**	132 (18)	90 (41)	**5.55 (2.82, 10.94)**
			P $_{trend}$ ***<0.000***			***P*** $_{trend}$ ***<0.000***			***P*** $_{trend}$ ***<0.000***
**When you were in your 30s with how many different partners did you have intercourse?**									
0-1	884 (56)	72 (19)	Ref.	509 (61)	35 (21)	Ref.	375 (51)	37 (17)	Ref.
2-9	540 (34)	216 (56)	**2.57 (1.85, 3.57)**	263 (31)	91 (55)	**2.69 (1.69, 4.29)**	277 (38)	125 (57)	**2.46 (1.53, 3.96)**
10 or more	143 (9)	97 (25)	**4.29 (2.87, 6.41)**	65 (8)	38 (23)	**3.74 (2.08, 6.73)**	78 (11)	59 (27)	**4.72 (2.68, 8.33)**
			P $_{trend}$ ***<0.000***			***P*** $_{trend}$ ***<0.000***			***P*** $_{trend}$ ***<0.000***
**Throughout your life, what is the total number of partners with whom you have had sexual intercourse?**									
<5	584 (37)	23 (6)	Ref.	337 (40)	10 (6)	Ref.	247 (33)	13 (6)	Ref.
5-19	647 (41)	157 (40)	**4.10 (2.46, 6.84)**	344 (41)	72 (42)	**5.44 (2.51, 11.80)**	303 (41)	85 (38)	**3.17 (1.59, 6.33)**
20 or more	352 (22)	217 (55)	**7.74 (4.63, 12.95)**	164 (19)	90 (52)	**9.96 (4.53, 21.91)**	188 (25)	127 (56)	**6.07 (3.05, 12.10)**
			P $_{trend}$ ***<0.000***			***P*** $_{trend}$ ***<0.000***			***P*** $_{trend}$ ***<0.000***

[a]Unconditional logistic regression adjusted for body mass index at study enrollment (BMI, kg m^-2), age at study entry, education (high school or less, some college, college, professional school), family history of prostate cancer (first degree relatives, yes/no), smoking history (never, former, current), condom use (usually use, yes/no), aspirin use (regular user, yes/no), IFNL4 rs368234815 genotype (ΔG/ΔG or ΔG/TT vs. TT/TT), and race
OR denotes Odds Ratio; 95% CI denotes 95% confidence interval
NOTE: Bolded data indicate significant associations in the multivariable logistic regression analysis.

Supplementary Table 3. IFNL4 rs368234815 allele and genotype frequencies

		Allele Frequency		Genotype Frequency		
		**TT** *N* **(%)**	**ΔG** *N* **(%)**	**TT/TT** *N* **(%)**	**ΔG/TT** *N* **(%)**	**ΔG/ΔG** *N* **(%)**
**African American**		**337 (38.3)**	**544 (61.7)**	**139 (16)**	**396 (45)**	**346 (39)**
	Control	178 (38.9)	279 (61.1)	71 (15)	214 (47)	172 (38)
	Case	159 (37.5)	265 (62.5)	68 (16)	182 (43)	174 (41)
**European American**		**596.5 (66.3)**	**303.5 (33.7)**	**407 (45)**	**379 (42)**	**114 (13)**
	Control	327.5 (66.1)	168.5 (33.9)	226 (46)	203 (41)	67 (13)
	Case	269 (66.6)	135 (33.4)	181 (45)	176 (43)	47 (12)

Supplementary Table 4. Association between number of sexual partners and prostate cancer risk in men with one copy vs. two copies of the risk variant

	**Total**			**IFNL4 TT/TT**			**IFNL4 ΔG/TT**			**IFNL4 ΔG/ΔG**		
	**Control** ***N*** **(%)**	**Case** ***N*** **(%)**	**Multivariable[a] OR (95% CI)**	**Control** ***N*** **(%)**	**Case** ***N*** **(%)**	**Multivariable[b] OR (95% CI)**	**Control** ***N*** **(%)**	**Case** ***N*** **(%)**	**Multivariable[b] OR (95% CI)**	**Control** ***N*** **(%)**	**Case** ***N*** **(%)**	**Multivariable[b] OR (95% CI)**
**When you were in your teens with how many different partners did you have intercourse?**												
0-1	444 (46)	347 (38)	Ref.	152 (54)	96 (43)	Ref.	161 (41)	127 (38)	Ref.	89 (40)	59 (29)	Ref.
2 or more	524 (54)	555 (62)	1.09 (0.87, 1.36)	130 (46)	129 (57)	1.20 (0.80, 1.80)	227 (59)	206 (62)	0.89 (0.63, 1.26)	132 (60)	145 (71)	1.35 (0.86, 2.12)
**When you were in your 20s with how many different partners did you have intercourse?**												
0-1	308 (31)	243 (26)	Ref.	96 (33)	78 (32)	Ref.	121 (30)	81 (23)	Ref.	60 (26)	35 (16)	Ref.
2 or more	693 (69)	707 (74)	1.20 (0.95, 1.52)	196 (67)	167 (68)	0.83 (0.55, 1.25)	282 (70)	268 (77)	1.37 (0.96, 1.97)	167 (74)	178 (84)	**1.69 (1.01, 2.80)**
**When you were in your 30s with how many different partners did you have intercourse?**												
0-1	544 (54)	412 (43)	Ref.	172 (59)	131 (53)	Ref.	215 (53)	144 (41)	Ref.	111 (49)	67 (31)	Ref.
2 or more	457 (46)	539 (57)	**1.44 (1.15, 1.79)**	121 (41)	115 (47)	1.05 (0.70, 1.57)	187 (47)	205 (59)	**1.62 (1.16, 2.27)**	116 (51)	146 (69)	**1.86 (1.17, 2.95)**
**Throughout your life, what is the total number of partners with whom you have had sexual intercourse?**												
<5	347 (34)	260 (27)	Ref.	113 (39)	84 (34)	Ref.	131 (32)	90 (26)	Ref.	68 (29)	41 (19)	Ref.
5-19	416 (41)	388 (40)	1.14 (0.89, 1.46)	110 (38)	92 (37)	0.96 (0.61, 1.49)	184 (44)	144 (41)	1.10 (0.75, 1.60)	92 (39)	87 (40)	1.64 (0.95, 2.85)
20 or more	254 (25)	315 (33)	**1.48 (1.11, 1.96)**	70 (24)	70 28)	0.99 (0.59, 1.68)	98 (24)	119 (34)	**1.64 (1.06, 2.53)**	73 (31)	89 (41)	**1.97 (1.11, 3.53)**
			**P_{trend} 0.006**			P_{trend} 0.966			**P_{trend} 0.023**			**P_{trend} 0.027**

[a]Unconditional logistic regression adjusted for body mass index at study enrollment (BMI, kg m^-2), age at study entry, education (high school or less, some college, college, professional school), family history of prostate cancer (first degree relatives, yes/no), smoking history (never, former, current), condom use (usually use, yes/no), aspirin use (regular user, yes/no), race, and IFNL4 rs368234815 genotype (ΔG/ΔG or ΔG/TT vs. TT/TT)

[b]Unconditional logistic regression adjusted for body mass index at study enrollment (BMI, kg m^-2), age at study entry, education (high school or less, some college, college, professional school), family history of prostate cancer (first degree relatives, yes/no), smoking history (never, former, current), condom use (usually use, yes/no), aspirin use (regular user, yes/no), and race

OR denotes Odds Ratio; 95% CI denotes 95% Confidence interval

NOTE: Bolded data indicate significant associations in the multivariable logistic regression analysis.

Supplementary Table 5. Expression values for SeV, IFNL4, and ISG15 used for plotting Supplementary Figure 1

		PC3	DU145
	**SeV infection**	**Normalized dCt values, log 2 scale**	**Normalized dCt values, log 2 scale**
**SeV RNA**	-	-18.234	-18.202
		-18.126	-18.499
		-18.243	-18.47
	+	2.039	1.614
		1.708	1.517
		1.939	1.325
**IFNL4 mRNA**	-	-16.812	-17.239
		-16.589	-17.788
		-17.175	-17.388
	+	-7.583	-13.187
		-7.522	-13.151
		-7.249	-13.141
**ISG15 mRNA**	-	-3.65	-4.531
		-3.642	-4.867
		-4.037	-4.897
	+	0.862	-0.184
		0.829	-0.437
		1.169	-0.199

Supplementary Table 6. Association between gonorrhea and prostate cancer risk stratified by IFNL4 rs368234815 genotype

	Total				IFNL4- ΔG/ΔG or ΔG/TT				IFNL4- TT/TT			
	**Control** *N* **(%)**	**Case** *N* **(%)**	**Univariable OR (95% CI)**	**Multivariable[a] OR (95% CI)**	**Control** *N* **(%)**	**Case** *N* **(%)**	**Univariable OR (95% CI)**	**Multivariable[b] OR (95% CI)**	**Control** *N* **(%)**	**Case** *N* **(%)**	**Univariable OR (95% CI)**	**Multivariable[b] OR (95% CI)**
**Did a doctor ever tell you that you had gonorrhea?**												
No	862 (83)	750 (77)	Ref.	Ref.	521 (79)	430 (74)	Ref.	Ref.	269 (91)	202 (81)	Ref.	Ref.
Yes	172 (17)	226 (23)	1.51 (1.21, 1.88)	**1.33 (1.02, 1.73)**	135 (21)	149 (26)	1.34 (1.03, 1.74)	1.27 (0.93, 1.73)	28 (9)	47 (19)	2.24 (1.35, 3.69)	**1.81 (1.02, 3.19)**

[a]Unconditional logistic regression adjusted for body mass index at study enrollment (BMI, kg m^-2), age at study entry, education (high school or less, some college, college, professional school), family history of prostate cancer (first degree relatives, yes/no), smoking history (never, former, current), condom use (usually use, yes/no), aspirin use (regular user, yes/no), race, and IFNL4 rs368234815 genotype (ΔG/ΔG or ΔG/TT vs. TT/TT)

[b]Unconditional logistic regression adjusted for body mass index at study enrollment (BMI, kg m^-2), age at study entry, education (high school or less, some college, college, professional school), family history of prostate cancer (first degree relatives, yes/no), smoking history (never, former, current), condom use (usually use, yes/no), aspirin use (regular user, yes/no), and race

OR denotes Odds Ratio; 95% CI denotes 95% Confidence interval

NOTE: Bolded data indicate significant associations in the multivariable logistic regression analysis.
