# Supplementary material for: IFNL4-ΔG is associated with prostate cancer among men at increased risk of sexually transmitted infections
Source: Commun Biol. 2018 Nov 14;1:191. doi: 10.1038/s42003-018-0193-5 (PMC6235841; doi:10.1038/s42003-018-0193-5)
Supplement: Supplementary file 2 — Description of additional supplementary items [file 42003_2018_193_MOESM2_ESM.docx]

Description of additional supplementary items

**Supplementary Data 1:** *IFNL4* genotype and clinical information for 976 cases and 1034 controls. The table is provided in Excel format and includes individual-level case/control status, ethnicity/race, *IFNL4* genotype, Gleason score, Stage, and PSA measurement (ng per ml).
